# What Is Behind? Impact of Pelvic Pain on Perceived Stress and Inflammatory Markers in Women with Deep Endometriosis

**DOI:** 10.3390/jcm13102927

**Published:** 2024-05-16

**Authors:** Jordana Diniz Osaki, Marco Aurelio Pinho Oliveira

**Affiliations:** 1 Núcleo Avançado de Endometriose e Robótica, Hospital DF Star, Brasília 70390-140, Brazil; 2 Department of Gynecology, State University of Rio de Janeiro, Rio de Janeiro 21941-617, Brazil; endometriose@gmail.com

**Keywords:** endometriosis, pelvic pain, chronic pain, inflammation, stress, women’s health

## Abstract

**Introduction/Objectives:** Endometriosis affects 10% of women worldwide. It is noteworthy that this condition is often accompanied by pelvic pain and stress. Endometriosis is a debilitating gynecological condition where tissue similar to the uterine lining grows outside the uterus, often causing significant pain and reproductive issues. We aimed to study the relationship between the intensity of pelvic pain, and stress and inflammatory markers in women with deep endometriosis. **Methods:** This cross-sectional study analyzed women diagnosed with deep endometriosis through imaging, surgery, and/or biopsy. We assessed pain using the Numerical Rating Scale (NRS). Stress was assessed with the Perceived Stress Scale (PSS-10) questionnaire and the serum cortisol levels. Additionally, we analyzed inflammatory markers, including C-reactive protein (CRP) and erythrocyte sedimentation rate (ESR). **Results:** Fifty-two women, with an average age of 37.8 ± 6.9 years, participated in this study. Forty-four percent of these participants demonstrated high levels of stress, as indicated by scores above 26 on the PSS-10. Those categorized with “high stress” on the PSS-10 questionnaire exhibited significantly higher pain levels compared to those with “low stress” (*p* < 0.05). Furthermore, patients experiencing more-severe pelvic pain (pain score > 7) had notably higher serum cortisol levels. Women with intense pelvic pain (scores above 7 on the NRS) had significantly elevated serum cortisol levels (Cohen’s d = 0.72; *p* = 0.018). **Conclusions:** A positive association was found between stress levels and the intensity of pelvic pain in women with deep endometriosis, suggesting an interconnection between emotional aspects and biological responses.

## 1. Introduction

Endometriosis, a prevalent yet complex gynecological condition, manifests through the growth of tissue resembling the endometrium outside the uterine cavity. This tissue can be found in various locations including, but not limited to, the ovaries, fallopian tubes, and the pelvic peritoneum. In more-severe cases, termed ‘deep endometriosis’, the endometrial-like tissue penetrates more than 5 mm into the peritoneal surface [1,2]. This form of the disease often involves key pelvic structures and is particularly associated with higher pain levels and greater impact on quality of life. Deep endometriosis is distinguished not only by its depth but also by its potential to significantly infiltrate the organs it affects, such as the bowel, bladder, and in rare instances, even the diaphragm [1,2]. These characteristics make it one of the most debilitating forms of the condition, often requiring more complex management strategies including advanced surgical interventions. Its symptoms are diverse, ranging from severe pelvic pain to infertility, significantly affecting women’s health and quality of life. The disease can localize variably, with lesions found on the ovaries, fallopian tubes, pelvic peritoneum, and even in more rare locations such as the lungs and nasal passages, which can result in a wide array of symptoms [1,2].

Epidemiological studies have helped quantify the socioeconomic impact and quality of life of women affected by endometriosis [2,3,4,5,6,7,8]. This research has revealed not only the substantial financial burden associated with endometriosis but also highlighted the importance of holistic approaches that consider not only medical aspects but also the social and psychological impacts of this condition. Moreover, endometriosis is defined by the abnormal growth of endometrial-like tissue, similar to the lining inside the uterus, but located outside the uterine cavity. This ectopic tissue behaves similarly to the endometrium, responding to hormonal changes of the menstrual cycle, which often leads to chronic inflammation and scarring. As a result, individuals with this condition frequently experience debilitating symptoms, most notably chronic pelvic pain. This pain can be exacerbated during menstrual periods and can significantly impair daily activities and quality of life [3,8,9,10,11]. The treatment strategies for endometriosis are equally varied, encompassing pain management, hormonal therapies, and surgical options aimed at removing endometrial implants. Despite these measures, endometriosis remains a challenging condition to manage due to its chronic nature and the intricate link between its physical manifestations and psychological impacts [1]. This burden affects approximately 10% of women globally and represents a significant concern for women’s health [2,3,4]. This condition has been the subject of intensive study, and significant advancements have been made in understanding it [5,6,7].

Gruber et al. (2021) have sought to elucidate the variable and often intense nature of the pain experienced by affected women [11]. This scenario reflects the complexity of pain associated with endometriosis, which may involve different aspects such as dysmenorrhea, dyspareunia, non-menstrual pelvic pain, and dysuria [12]. Additionally, recent studies have explored correlations between pain intensity and inflammatory markers, highlighting the potential contribution of chronic inflammatory processes to pain severity [6,13,14,15]. Furthermore, another potential factor that may influence pain intensity and/or symptoms in women with endometriosis is perceived stress. It is known that stress has a close relationship with inflammation and appears to be associated with higher pain intensities.

However, the link between endometriosis and perceived stress is a complex and sometimes conflicting field of study. Understanding this interaction between endometriosis and stress is crucial for providing more comprehensive insights into the impacts of the condition on the mental health of affected women. Pain is a central manifestation of endometriosis, and its intensity can vary considerably among affected women [6,9,10,11,16]. Additionally, endometriosis is associated with a chronic inflammatory state, suggesting a possible link between pain and inflammatory processes [6,16]. 

Despite growing knowledge about the pathophysiological pathways, both phenomena are not yet fully understood. Although there have been important advances in understanding endometriosis, gaps in knowledge still persist, especially concerning the interaction between pain, stress, and inflammatory markers. The objective of the present study is to evaluate whether women with deep endometriosis and high levels of stress and/or inflammation have more pain than women with deep endometriosis and low levels of stress and/or inflammation. We hypothesize that patients with deep endometriosis and high levels of stress, experience a greater pain intensity than patients with low levels of stress.

## 2. Materials and Methods

### 2.1. Study Design and Participants

The research protocol was approved by the Human Research Ethics Committee of the Pedro Ernesto University Hospital of the State University of Rio de Janeiro (number: 5.524.346, CAAE: 58289421.0.0000.5259). In this study, all participants were recruited prior to undergoing surgical procedures. Each participant underwent imaging techniques, such as ultrasound or MRI, to identify potential candidates for surgical treatment. This pre-operative recruitment strategy was essential for ensuring that each patient’s diagnosis of deep endometriosis could be surgically confirmed, thus meeting the study’s inclusion criteria. Moreover, all patients were hormonally blocked with estrogen and progesterone medications to manage symptoms and prevent disease progression. This uniform treatment approach ensured consistency in the study and minimized the variability in disease activity.

This manuscript was prepared in accordance with the STROBE checklist guidelines for observational reports. This cross-sectional study was conducted at the Gynecology Outpatient Clinic of Hospital Santa Luzia and DF Star, located in Brazil, and at the Endometriosis Outpatient Clinic of the Pedro Ernesto University Hospital.

We invited participants to join the study and provided clarifications about its voluntary nature, objectives, and assurances regarding the anonymity and confidentiality of the information provided. Before participation, the Informed Consent Form was presented for reading, understanding, and signing, thus authorizing inclusion in the research.

To participate in the study, candidates had to meet the following inclusion criteria: aged between 18 and 45 years, and a diagnosis or strong clinical suspicion of deep endometriosis based on imaging techniques, such as ultrasound or MRI. All participants in this study were definitively diagnosed with deep endometriosis, either through imaging techniques such as ultrasound or MRI that indicated the presence of infiltrative endometriosis nodules of 2 cm or more, or through surgical procedures. To ensure the specificity of our diagnosis and to minimize the risk of including false positives, every case initially identified through imaging was confirmed by histopathological analysis following surgery. This approach allowed us to establish a robust diagnosis of deep endometriosis in all study participants, thereby reducing potential diagnostic bias. The inclusion solely of patients with histologically confirmed deep endometriosis ensures that our study accurately reflects the characteristics and implications of this specific and severe form of the disease.

### 2.2. Data Collection Procedures

After participant selection, information was collected through structured interviews, medical record reviews, and analysis of imaging examination results. Data collection included demographic information, medical history, characteristics of endometriosis, assessments of pain and stress, and associated biomarkers.

### 2.3. Pain

Pain intensity in our study participants was quantitatively assessed using the Numerical Pain Scale (NRS), a widely accepted instrument in clinical research for evaluating pain levels. The NRS ranges from 0 to 10, where 0 indicates ‘no pain’ and 10 represents ‘the worst possible pain’. This scale allows patients to subjectively rate their pain, providing a straightforward and effective measure of the severity of their symptoms. This tool was validated for this population by previous studies [17,18,19,20]. This assessment was performed for each of the characteristics of pelvic pain, including dyspareunia, dysmenorrhea, non-menstrual pelvic pain, dysuria, or dyschezia. The score can either be used as a numerical variable or dichotomized as a categorical variable. We dichotomized the data, stratifying patients into groups with severe pain and those with mild/moderate pain [21].

### 2.4. Stress Markers

For the assessment of psychological stress, we used the 10-item Perceived Stress Scale (PSS-10) [22]. A confirmatory factor analysis (CFA) conducted by Taylor (2015) concluded that a two-factor model best describes the Perceived Stress Questionnaire (PSS-10), covering the dimensions of perceived helplessness and lack of self-efficacy. As previous studies have described, individuals must indicate how often they felt or thought in a certain way over the past month for each question. The scale ranges from 0 (never) to 4 (very often). The score ranges from 0 to 40, and as the sum of the score increases, so does the level of stress. We dichotomized patients into a low-stress group (score ≤ 26) and high-stress group (score > 26) [19]. Serum cortisol (human cortisol kit, SIEMENS Medical Solutions Diagnostics, USA, chemiluminescence method) was used as a biochemical marker for stress.

### 2.5. Inflammatory Markers

All samples were collected in the morning and analyzed by a single clinical analysis laboratory with certification (Sabin)—Laboratory Clinical Accreditation Program—PALC and ISO 9001, to achieve greater precision in the results. All analysis occurred between 8–9 am for all patients. The inflammatory markers examined in this study included C-reactive protein and the erythrocyte sedimentation rate (ESR).

### 2.6. Statistical Analysis

Descriptive data are presented using the mean and standard deviation for continuous variables, and frequency and percentage for categorical variables. For comparison between independent groups, the independent t-test was used. To analyze effect size, Cohen’s d was used. Effect sizes of 0.2, 0.5, and 0.8 are considered small, medium, and large, respectively. For all analyses, the level of significance adopted was *p* < 0.05. For database organization, Microsoft Excel^®^ 2016 version 16.0 software was used, and for data processing, the JAMOVI version 2.2.5 software was used.

## 3. Results

Table 1 presents the continuous descriptive data, and Table 2 reflects the categorical data in frequency and percentage. Table 1 shows that the sample is composed of women with an average age of 38 ± 7 years and with an average BMI of 24.07 ± 2.49 kg/m^2^. Table 2 also shows that 42% of the women have given birth, with 47% of these births being cesarean deliveries, and 60% of the women are nulliparous. All the women reported pain (primary complaint in 82%, and only 18% reported complaints of infertility). About 45% of the women were categorized as having a high level of stress (PSS score > 26).

In Table 3, we present data comparing pain intensity at different levels of stress. This analysis reveals a positive association between stress levels and pain levels; in all comparisons, patients classified as having “high stress” exhibited higher levels of pain compared to those with “low stress” (*p* < 0.05).

**Figure 1:**
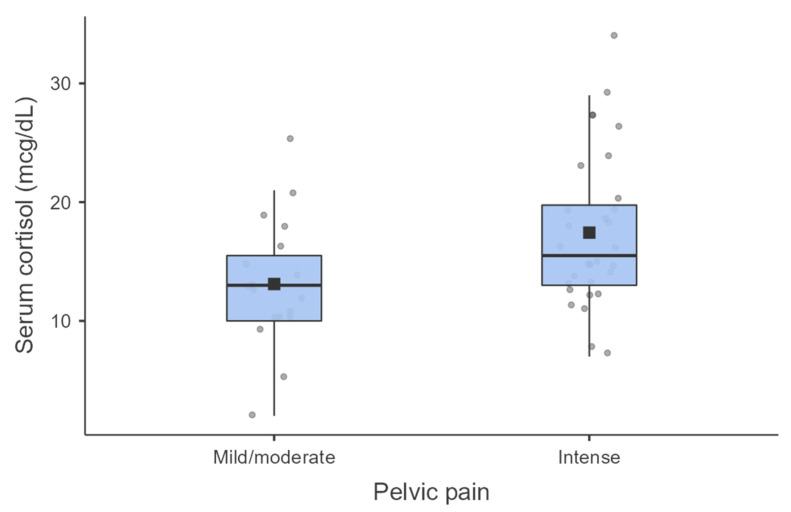
Comparison of Serum Cortisol Levels Among Groups with Different Levels of Pelvic Pain.

In Table 4, our analysis of cortisol, CRP, and ESR levels in groups based on the intensity of pelvic pain revealed significant differences. These differences indicate higher levels of serum cortisol in patients with intense pelvic pain (NRS > 7) (Cohen’s d = 0.72; *p* = 0.018). Figure 1 shows a comparison of serum cortisol levels between groups with severe pain and mild/moderate pain. 

## 4. Discussion

Our study reveals a significant relationship between elevated stress levels and increased intensity of pelvic pain among women with deep endometriosis [23,24,25,26]. This correlation suggests that stress exacerbates the painful symptoms of endometriosis, which may also manifest in related symptoms such as dyspareunia, dyschezia, strangury, and low back pain. Elevated cortisol levels, a marker of both stress and inflammation, were found in women experiencing more-severe pain, indicating a bi-directional interaction where stress enhances inflammatory responses, which in turn contribute to the severity of pain [23,24,25,26,27].

Cortisol plays a crucial role in modulating inflammation and directly influencing pain intensity [28,29,30]. Elevated cortisol levels may disrupt normal immune system regulation, intensifying the chronic inflammation commonly associated with endometriosis [12,13]. The detection of increased cortisol levels in patients with intense pelvic pain underscores cortisol’s dual role as both a stress biomarker and a modulator of inflammation, reinforcing the need for treatments that address both hormonal and inflammatory aspects of the disease [13,14].

The positive correlation between perceived stress levels and pain intensity suggests that interventions aimed at reducing stress could significantly alleviate pain [25,29,30,31]. Psychological factors are thus significant in managing endometriosis pain, highlighting the potential of psychosocial interventions to improve overall well-being in women with endometriosis [20]. Integrating psychological therapies with traditional medical treatment could lead to better patient outcomes by addressing both the psychological and physiological aspects of endometriosis.

A significant limitation of our study is its cross-sectional design, which restricts our ability to infer causality between the observed variables—specifically, the relationships among endometriosis, pelvic pain, stress, and inflammatory markers. Cross-sectional research provides a snapshot of correlations but does not allow for determination of which variable precedes or potentially causes another. For example, while higher levels of stress and increased inflammatory markers are associated with more-severe pelvic pain, we cannot conclusively determine whether stress and inflammation lead to increased pain or if pain itself triggers stress and inflammatory responses. Without longitudinal data, it remains unclear whether interventions that reduce stress or inflammation can lead to sustained reductions in pain, or how these variables interact over time. The absence of longitudinal measures prevents us from observing the progression of symptoms, and potential fluctuations in stress levels and inflammatory markers over the course of the disease. This limitation underscores the need for future studies with a longitudinal design that can track changes in these variables over time, providing more definitive evidence of causality and helping to clarify the directionality of the relationships observed. Such studies could greatly enhance our understanding of endometriosis, leading to more effective interventions targeting the specific mechanisms driving the disease process.

Despite these limitations, the findings of this cross-sectional study are valuable as they highlight significant associations that warrant further investigation and could inform more comprehensive approaches in both the research and clinical management of endometriosis. Elevated stress levels and associated inflammatory responses may significantly exacerbate the pain experienced by these patients. The observed correlations underscore the necessity of adopting an integrative treatment approach that addresses both psychological and physiological aspects of endometriosis. By incorporating stress management and anti-inflammatory strategies into the therapeutic regimen, clinicians may improve patient outcomes. Moving forward, longitudinal studies are crucial to establish the causality and directionality of these relationships, paving the way for targeted interventions that mitigate pain and enhance the quality of life for those affected by endometriosis.

## 5. Conclusions

In conclusion, our study provides significant insights into the relationship between stress, inflammation, and pain in deep endometriosis. Notably, we identified that high cortisol levels, indicative of both stress and inflammation, were specifically associated with increased pain in the domains of dysmenorrhea, dyspareunia, and non-menstrual pelvic pain. These findings emphasize that in women with deep endometriosis, the exacerbation of pain symptoms is closely linked to elevated stress and inflammatory markers.

This delineation is vital for clinical practice, as it guides targeted treatment strategies that are appropriately tailored to the severity and specific characteristics of the endometriosis being treated. Future research should continue to explore these relationships in other forms of endometriosis to fully understand the broader applicability of our findings. Ultimately, enhancing our understanding of these mechanisms can lead to more effective interventions that improve the management and quality of life for all women suffering from this debilitating condition.

## Figures and Tables

**Table 1:** Descriptive Data.

Variables	Mean	SD
Age (years)	37.81	6.9
Height (cm)	166.84	6.04
BMI (kg/m^2^)	24.07	2.49
Menarche (age)	11.21	0.99
Pain (months)	16.94	8.37
Pelvic pain	7.19	2.86
Lower back pain	5.87	3.40
Dysmenorrhea	7.81	2.87
Dyspareunia	5.91	2.91
Dyschezia	3.60	3.09
Strangury	2.02	2.45

**Table 2:** Frequency and Percentage of Sample Characteristics.

Categorical Variables	n	%
Births		
0	29	58%
1 or more	21	42%
Cesarean Deliveries		
0	27	53%
1	24	29%
2	9	18%
Abortions		
0	40	82%
1 or 2	9	18%
Ethnicity		
White	36	75%
Latin/Black	12	25%
Marital Status		
Married	40	80%
Single	10	20%
PSS Score		
Low stress (≤26)	29	56%
High stress (>26)	23	44%

**Table 3:** Comparison of Pain Level at Different Stress Levels.

Pain Symptoms	Low	NRS	High	NRS	*p*	ES
	Stress (*n*)	(Mean; SD)	Stress (*n*)	(Mean; SD)		
Dysmenorrhea	21	6.7; 3.3	15	9.2; 1.5	0.017	0.85
Dyspareunia	24	4.96; 3.10	19	7.11; 2.18	0.014	0.78
Strangury	29	1.07; 1.89	23	3.22; 2.58	0.001	0.97
Dyschezia	29	1.93; 2.64	23	5.70; 2.22	<0.001	1.52
Lower Back Pain	29	4.38; 3.23	23	7.74; 2.61	<0.001	1.13
Pelvic Pain	29	6.14; 3.14	23	8.52; 1.78	0.002	0.91

ES: Effect Size; NRS (Numerical Rating Scale).

**Table 4:** Analysis of Cortisol, CRP, and ESR Levels According to Pelvic Pain Levels.

Variables	Mild/Moderate Pain (n)	NRS (Mean; SD)	Severe Pain (n)	NRS (Mean; SD)	*p*	ES
Serum Cortisol	19	13.11; 5.35	30	17.43; 6.44	0.018	0.72
CRP	21	9.52; 15.86	30	10.73; 29.72	0.866	0.05
ESR	20	3.75; 4.47	30	7.07; 15.71	0.364	0.26

ES: Effect Size; NRS: Numeric Rating Scale; ESR: Erythrocyte sedimentation rate.

## Data Availability

Data are available upon reasonable request to the corresponding author.
